# Editorial: Nanomaterials Mediated Immunomodulation in Cancer: Current Perspective from Bench to Clinic

**DOI:** 10.3389/fimmu.2026.1784752

**Published:** 2026-01-26

**Authors:** Rohit Kumar Tiwari, Syed Mohd Danish Rizvi

**Affiliations:** 1 Department of Clinical Research, Sharda School of Allied Health Sciences, Sharda University, Greater Noida, Uttar Pradesh, India; 2 Department of Pharmaceutics, College of Pharmacy, University of Ha’il, Ha’il, Saudi Arabia

**Keywords:** biosensing techniques, drug delivery systems, immunomodulation, nanoparticles, neoplasms

Over the last few decades, nanotechnology has moved from the periphery of the biomedical sciences to the centre of modern-day immunology. Nanotechnology, initially considered an effective approach for improving drug delivery, has now evolved into a more advanced field focused on understanding how various nanomaterials can influence, crosstalk with, and reprogram immune responsiveness. Indeed, the contributions in the current Research Topic, spanning cancer biology, diagnostics, infectious disease immunology, and systemic regulatory mechanisms, collectively indicate how nanoparticles are redefining our understanding of their role in immune modulation. Across the articles published in this Research Topic, a prominent theme is the ability of nanoparticles to modulate immune processes with an accuracy that mimics natural immunological interactions. In hepatocellular carcinoma, green synthesized *Sphaeranthus indicus* silver nanoparticles demonstrated not only considerable cytotoxic effects but also induced apoptosis, and one of the bioactive components of *S. indicus* was hypothesized to modulate the BRD4 proteins (Konduskar et al.). BRD4 is a member of Bromodomain and Extra-Terminal (BET) family and acts as an epigenetic regulatory switch that binds specifically to acetylated histones and influences transcription through regulation of RNA polymerase II phosphorylation (1). This observation supports the growing recognition that nanoparticles may function as active immunomodulatory agents, with the potential to influence tumor cell death, immune responsiveness, and inflammatory signaling within the tumor microenvironment. Diagnostics is another critical area where the immunological insights provided by certain nanomaterials are proving to be crucial. Peptide-functionalized nanoparticle-based biosensors have further highlighted that how nanoparticles facilitate the immune recognition more comprehensively at clinical level. It has been shown that presentation of engineered epitopes derived from SPON2 (a pathogen recognition molecular pattern) and MSMB (potential biomarker of prostate cancer) achieves elevated diagnostic performances by converting highly subtle antigen-antibody interaction into substantially sensitive detection signals for diagnostic purposes (Ye et al.). A similar nanomaterial-aided immunodiagnostic framework has been reported for hepatocellular carcinoma by leveraging epitope-level mapping and multiomics profiling to subsequently enable the detection of highly sensitive immune markers associated with tumor progression (Luo et al.).

These reports have thus clearly outlined the indispensable role of nanoparticles in deciphering the immune landscape in different cancers, predominantly during the early stages when the signals are faint and commonly missed by conventional assays. Nanotherapeutics have also shown their relevance as potential modulators of pivotal immune signalling nexus. Structure and interaction-based modelling of certain important biomarkers such as Epidermal Growth Factor Receptor (EGFR), anaplastic lymphoma kinase (ALK), Kirsten rat sarcoma viral oncogene homolog (KRAS) and programmed cell death protein-1 (PD-1) have shown that nanoparticles can be utilized to interact with key points of immune regulation in lung carcinoma (Peng et al.). Indeed, by further increasing their specificity, these nanotherapeutics can be proven effective in clinically modulating the immune checkpoint pathways, sensitizing the tumor cells towards specific immunotherapies and alter the signalling pathways and kinetics. Transitionally, virus-like nanoparticles (VLPs) evaluated in competent cancer animal model has been reported to be efficacious in inciting cytokine responses and reprogramming the tumor microenvironment, thus proving their therapeutic role (Singh et al.).

This indeed is indicative of the notion that nanoparticles directly modulate the immune responsiveness rather than being just a delivery vehicle. While these highly nanoparticle centric studies have highlighted exciting innovations, other contributions in this Research Topic provides essential insight into the regulatory environments in which these technologies must operate. Certainly, a major challenge in cancer immunotherapy remains to be the occurrence of suppressive immune environment that limits the anti-tumor response clinically. Tumor cells have a tendency to evade the host immune response by lowering the antigen exposure, reduced MHC expression and increasing the population of immunosuppressive cells and/or soluble factors that facilitates the suboptimal responses to immune checkpoint inhibitors (Zhang et al.). These areas represent the specific barriers that nanotherapeutic-based approaches may overcome by elevating antigen presentation, inducing programmed cell death and/or rewiring suppressive myeloid niches.

Likewise, insights into broader immunoregulatory networks from studies examining chronic disease are also important. The onset of immune suppression and/or immune unresponsiveness rarely results from a single pathway but is rather the result of complex crosstalk between various signaling pathways and effector molecules (Chen et al.; Baena et al.). This, in turn, provides a plausible explanation behind the failure of conventional immunotherapies and success of multifunctional nanoparticle based clinical management. These multifunctional nanoparticles are capable of delivering combinations inducing signals to the tumor microenvironment and can also concomitantly disrupt multiple regulatory signaling nexus. A mechanistic understanding of these regulatory pathways is crucial for designing next-generation of nanotherapeutics with intrinsic ability to navigate, overcome and/or directly remodel the immune suppressive environment within cancerous tissues.

Identification of certain shared regulatory pathways/signature molecules across different diseases is also a crucial task. Identification of these regulatory pathways/signature molecules may plausibly provide an exciting avenue for a unifying framework for envisaging disease outcomes, providing personalised therapeutic approach and/or designing immunomodulatory therapies with higher specificity (Singh et al.). Intriguingly, nanoparticles possess the ability to contribute in this directive. The properties of nanoparticles to remodel tissue microenvironment, crosstalk with immune receptors and subsequently transmit quantifiable molecular interaction signals make them powerful avenues for clinical mapping of immune states with high sensitivity.

Summarily, certain priorities have emerged for continuous advancements of nanoparticle mediated regulation of immune system in patients with different diseases. Firstly, improved integration of engineered nanomaterials with system immunology is essential to enable nanoparticles to navigate dynamic tissue microenvironments. Secondly, an enhanced mechanistic understanding of the interactions between nanotherapeutics and various immune cells is critical for refining the therapeutic targeting. Finally, translational studies, including those involving virus-like particles (VLPs), will definitely be invaluable in oncology for demonstrating real-world feasibility and safety. This Research Topic does not merely highlight past achievements in nanotechnology but instead emphasizes the future promise of the field. As the traditional boundaries between material science and immunology continue to dissipate, nanoparticles can undoubtedly play a transformative role in decoding immunological complexities and harnessing them for therapeutic benefits.
